# Baseline ECGs done in memory clinics in Leicestershire

**DOI:** 10.1192/bjo.2021.230

**Published:** 2021-06-18

**Authors:** Tamara Chithiramohan, Assad Kido, Mangesh Marudkar

**Affiliations:** Leicester Partnership Trust

## Abstract

**Aims:**

To ascertain the compliance of the Mental Health Service of Older People (MHSOP) memory clinics in obtaining ECGs, based on the agreed criteria.

**Background:**

Cholinesterase inhibitors are a main pharmacological treatment for Alzheimer's dementia (AD). These drugs may worsen pre-existing cardiac conditions or cause significant cardiac side effects. A baseline ECG can be beneficial before starting patients on these medications. Previously in Leicestershire, all memory clinic patients were receiving routine ECGs. However, new standards were set based on the NICE guidelines and criteria outlined in other regions, to reduce the use of this time consuming and expensive investigation for patients who may not require it.

**Method:**

A total of 120 patients attending memory clinics in Leicestershire over a 6 month period (April to September 2019), were randomly selected and their electronic records retrospectively reviewed. The data collection tool was designed to encompass the key aspects of the criteria for obtaining an ECG for those attending the memory clinic. The information was analysed using Microsoft Excel.

**Result:**

Of the 120 patients, 23 (19.2%) were diagnosed with AD, 10 (8.33%) with mixed and 19 (15.8%) with vascular dementia. 68 (56.7%) had a diagnosis of “other” which included mild cognitive impairment or diagnosis still under investigation. 0 patients were diagnosed with Lewy Body Dementia or Parkinson's dementia. Of the total number of patients, only 10 had an ECG done, 2 with a diagnosis of AD, 1 with mixed dementia, 1 with vascular dementia and 6 “other”. The 10 ECGs done were all requested by nursing staff.

Although 27 (22.5%) patients were identified to have a diagnosis of AD or mixed dementia, plus at least one of the criteria for an ECG, only 6 (22.2%) were discussed with the Multi-Disciplinary Team (MDT) following which only 3 of the 27 patients (11.1%) had an ECG

**Conclusion:**

Despite having clear criteria for requesting an ECG for those attending the memory clinic, compliance over the 6 month period was low. The following recommendations may be useful in improving compliance:

Displaying the ECG algorithm in the memory service clinic rooms.

Raise awareness amongst memory service clinicians of the criteria for requesting ECGs.

